# The enhanced effect and underlying mechanisms of mesenchymal stem cells with IL-33 overexpression on myocardial infarction

**DOI:** 10.1186/s13287-019-1392-9

**Published:** 2019-09-23

**Authors:** Yueqiu Chen, Jianfeng Zuo, Weiqian Chen, Ziying Yang, Yanxia Zhang, Fei Hua, Lianbo Shao, Jingjing Li, Yihuan Chen, Yunsheng Yu, Zhenya Shen

**Affiliations:** 1 Institute for Cardiovascular Science, Soochow University, Suzhou, China; 2 Department of Cardiovascular Surgery of the First Affiliated Hospital, Soochow University, Suzhou, China; 3 Nantong First People’s Hospital, Nantong, China

**Keywords:** IL-33, MSCs, Myocardial infarction, T cell, Macrophage cell

## Abstract

**Background:**

Interleukin 33 is known to have an important influence in the process of myocardial infarction, and the immunoregulatory function of MSCs could be influenced by cell factors. In this study, we evaluated the therapeutic efficacy of IL-33-overexpressing bone marrow mesenchymal stem cells (IL33-MSCs) on myocardial infarction (MI) and detected the inflammatory level and cardiac function in rats.

**Methods and results:**

First, we evaluated the proliferation of T cells and polarization of macrophages that had been co-cultured with Vector-MSCs or IL33-MSCs. Co-culture experiments indicated that IL33-MSCs reduced T cell proliferation and enhanced CD206^+^ macrophage polarization. Second, we determined the inflammation level and cardiac function of PBS-, Vector-MSC-, and IL33-MSC-injected rats. Echocardiography indicated that left ventricular ejection fraction (LVEF) was enhanced in IL33-MSC-injected rats compared with Vector-MSC-injected rats. Postmortem analysis of rat heart tissue showed reduced fibrosis and less inflammation in IL33-MSC-injected rats.

**Conclusion:**

These studies indicated that the IL33-MSC injection improved heart function and reduces inflammation in rats with MI compared with PBS or Vector-MSC injections.

**Graphical Abstract:**

IL-33 overexpression enhances the immunomodulatory function and therapeutic effects of MSCs on acute MI via enhancing the polarization of macrophages toward M2, enhancing the differentiation of CD4+ T cells toward CD4+IL4+Th2 cells, and finally, reducing heart inflammation and enhancing heart function. 
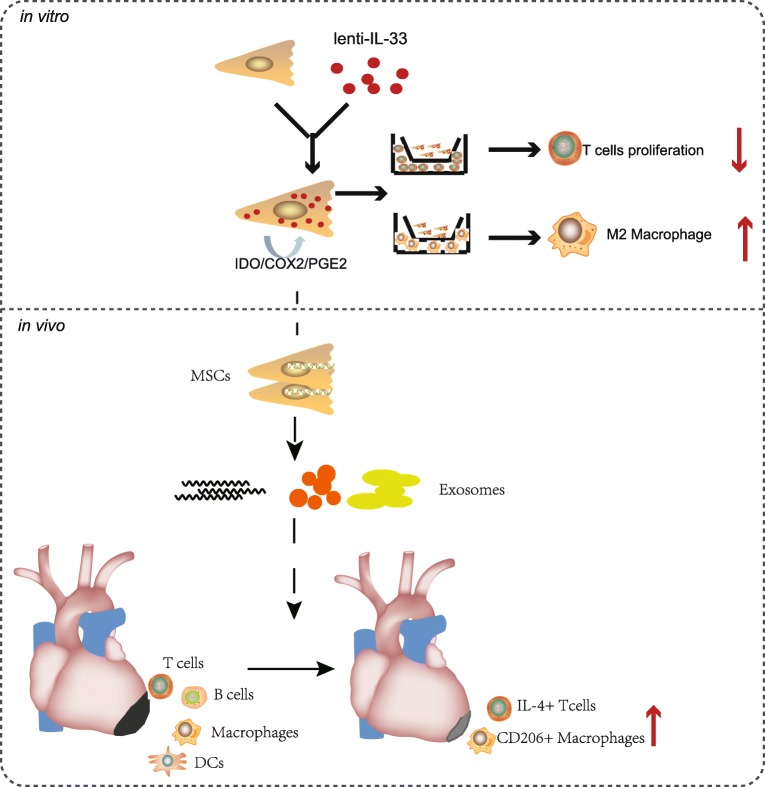

**Electronic supplementary material:**

The online version of this article (10.1186/s13287-019-1392-9) contains supplementary material, which is available to authorized users.

## Background

Cardiovascular disease is the leading cause of human death. An acute inflammatory response occurs following myocardial infarction, and the abnormal heart remodeling induced by strong inflammation is the main cause of heart failure [1]. Myocardial infarction (MI) is an important disease that leads to heart ischemia and myocardial death. There are three main phases after MI, including the inflammatory phase, the reparative and proliferative phases, and the maturation phase. The early inflammatory phase is complex and induces a strong innate immune response that mobilizes abundant neutrophils and macrophages to migrate to the myocardium, where they have important functions in determining the area of infarction [2, 3].

Mesenchymal stem cells (MSCs), a new cell therapy, have been shown to have huge potential for regenerative medicine and immunotherapy [4]. MSCs have widespread applications in cardiovascular therapy. For example, in patients with atherosclerosis, MSCs derived from skin can transform M1 macrophages secreting TNFα and IL-1β into M2 macrophages secreting IL-10 [5]. In clinical trials of patients with heart failure and an animal model of rats with heart failure, MSCs improved myocardial function in patients with severe ischemic heart failure and improved the ventricular function following stress-induced heart failure through an intramyocardial injection or coronary artery injection [6]. MSCs have certain immunoregulatory functions and have been used in many diseases [7, 8], but the current problem is how to enhance their immunoregulatory function. Biological materials and cell factors have emerged to increase the cardioprotective effects of MSCs; for example, MSCs coated with biological materials have stronger anti-inflammatory effects mediated through the chemotaxis function of CD45^+^ immune cells (neutrophil, granulocyte, macrophage) [9]. We found that overexpression of miR-133 in MSCs promoted their therapeutic efficacy in MI at both the inflammatory level and infarct size [10]. Accumulating evidence suggests that modification of MSCs with genes or microparticles can improve MSC-mediated immunosuppression [8, 11].

Interleukin 33 (IL-33), a recently described member of the IL-1 superfamily, is a multifunctional immune cytokines [12]. IL-33 participates in many inflammatory and immune reactions. IL-33 has important functions in tissue repair, organ transplantation, and maintenance of homeostasis through increasing proportions of Th2 and Treg cells [13]. IL-33 can act as an inhibitor of transcription pathways and as an alarm molecule for the immune system aimed at necrotic cells. Another function of IL-33 is as an immunomodulatory factor that regulates immune reactions [14]. In cardiovascular research, IL-33 plays vital warning and mitigation roles and has variable functions among different types of heart disease. For example, IL-33 can improve the survival rate of cardiomyocytes via the P38-MAPK signaling pathway and decrease the inflammation indexes by influencing the expression level of the pro-inflammatory factor HMGB1 in ischemia reperfusion, ultimately protecting cardiomyocytes [15]. IL-33 regulates the switch of T cells to Th1 or Th2 cells and the secretion of relative cytokines in atherosclerosis to reduce the formation of atherosclerosis plaque [16]. In cardiac xenotransplantation, IL-33 prolongs transplant survival time by improving the cell number of myeloid-derived suppressor cells and Treg cells [17].

In the present study, we investigated the therapeutic effect of MSCs overexpressing IL-33 in acute MI induced by ligation of the left anterior descending artery and the underlying mechanism. We found that IL-33 promoted the therapeutic efficacy of MSCs in MI by inducing macrophage polarization to the M2 phenotype and reducing proliferation. These findings indicated that IL-33 overexpression further enhanced the therapeutic efficacy of MSCs against MI and that the polarization of macrophages and T cells was necessary for the protective effects of MSCs. This study provides a novel therapeutic approach showing an improved therapeutic effect for MI.

## Materials and methods

### Animals

All animal experiments in this study were approved by the Ethics Committee of Soochow University (reference number: SZUM2008031233), and all experimental procedures were performed in accordance with protocols approved by the Institutional Animal Care and Usage Committee and conformed to the Guidelines for the Care and Use of Laboratory Animals published by the US National Institutes of Health. Rats were sacrificed by intra-peritoneal injection of pentobarbital (60 mg/kg). All male rats (4 2-week-old rats of used for the isolation of MSCs, 20 2-day-old rats used for the isolation of cardiomyocytes and fibroblasts, 2 2-week-old rats used for the isolation of bone marrow-derived monocyte-macrophages, 20 8-week-old rats used for time point experiments, and 40 8-week-old rats used for surgical and therapy experiments) were purchased from Model Animal Research Center of Nanjing University.

### Isolation and characterization of bone marrow-derived mesenchymal stem cells (MSCs)

Bone marrow-derived MSCs were isolated from 2-week-old Sprague-Dawley (SD) rats as previously described [10]. In brief, femurs and tibias from 2-week-old rats were used to collect the total bone marrow and were then subjected to treatment with red blood cell lysis buffer two times for 5 min at room temperature and washed with PBS. Afterwards, the cells were cultured in Dulbecco’s modified Eagle medium: nutrient mixture F-12 (DMEM/F12) with 10% fetal bovine serum (FBS) and 1% penicillin-streptomycin at 37 °C with 5% CO_2_. After 4–6 days of culturing, spindle cells reached 80% confluence and were passaged. Passage 3 cells were identified by flow cytometry with the following antibodies: anti-rat CD105-PE (12-1051, eBioscience), anti-rat CD73-PE (551123, BD), anti-rat CD90-PE (551401, BD), anti-rat CD29-FITC (555005, BD), anti-rat CD45-PE (6828, BD), and anti-rat CD11b/c-FITC (b141556, Biolegend). Passage 3 cells were used for the following experiments.

### Isolation and hypoxia conditioning of cardiomyocytes and fibroblasts in vitro

The hearts were excised from 2-day-old neonatal SD rats and then minced into 0.5–1-mm^3^ patches with sterile scissors. The pieces were digested with 1 mg/ml collagenase II for 15 min at 37 °C three times in PBS. The cells collected were seeded in 60-mm plates coated with gelatin (g5384, Sigma) in DF12 medium with 10% FBS. The un-adhered cells were transferred onto another plate coated with matrigel (Corning, 356,231) after 1 h. On the next day, the culture medium was switched, and beating myocardial cells (CMs) were observed 24 h later. Apart from the un-adhered cells, most of the adhered cells were fibroblasts, and after passaging two to three times with DF12 + 10%FBS, the spindle-shaped cells were fibroblasts (HFs).

For hypoxic culture, cells (CMs, HFs, and MSCs) were cultured in a tri-gas incubator (Thermo Fisher Scientific, Marietta, OH, USA) composed of 94% N_2_, 5% CO_2_, and 1% O_2_ with the same culture medium (DF12 and 10% FBS) for 24 h. Cells were then harvested for RT-qPCR analysis of IL-33 levels.

### Quantitative real-time PCR analysis

Total RNA was isolated from CMs, fibroblasts, MSCs, or heart tissues using TRIzol™ reagent (Invitrogen, USA) as described previously [4, 10]. Subsequently, RNA (500 ng) was reverse-transcribed into single-stranded cDNA using a PrimeScript™ RT reagent Kit (Takara, RR037A). The relative expression levels of genes were analyzed using SYBR Premix Ex Taq (Takara, RR42LR) in the ABI Step One-Plus Detection system (Applied Biosystems™, USA) according to the manuscript’s introductions. The primers used in this study are presented in Table 1. The relative gene expression levels were calculated using the 2^-△△CT^ method with GAPDH as an internal standard. Each assay was performed in triplicate biologically.

**Table 1:** Primer sequences for real-time PCR

Gene names	Forward primer sequences	Reverse primer sequences
*IL-33*	TGACACACTGAGTATCCAAGG	TATCTTTTTCTTGGTTTTTCC
*IDO*	ATCTTGCCGTTCCCTACT	CTGCGATTTCCACCATTA
*COX2*	CCTTCCTCCTGTGGCTGAT	GGGCAAAGAATGCGAACA
*T-bet*	AGCCGTTTCTACCCCGACCTT	GCAGAGGGTAGGAGTGTGGGC
*GATA-3*	CTCCTCCTCTACGCTCCTTGC	AAAAAAAAAGGAGGGAGAGA
*FOXP3*	CGAGCTTCCCAGAGAGAGAGT	TGAGTTGTTGCCTGCCCCTCA
*IL-6*	CCTTCTTGGGACTGATGT	ACTGGTCTGTTGTGGGTG
*TNFα*	CCACGCTCTTCTGTCTACTG	GCTACGGGCTTGTCACTC
*IL-1β*	TGTGATGTTCCCATTAGAC	AATACCACTTGTTGGCTTA
*iNOS*	CGGTGCGGTCTTTTCCTATG	GAGGGGAGATGATGTGAGGG
*Arg-1*	GACATCAACACTCCGCTGAC	TTGCCAATTCCCAGCTTGTC
*GAPDH*	TTC TTG TGC AGT GCC AGC CTC GTC	TAG GAA CAC GGA AGG CCA TGC CAG

### Plasmid construction and lentivirus preparation

The vector pCDH-CMV-MCS-EF1-copGFP was used as a backbone plasmid to reconstruct the lentiviral vector containing IL-33. Rat IL-33 mRNA was amplified from mRNA using a reverse transcript kit (Takara, RR037A) and PrimerSTAR HS DNA polymerase (Takara, R010A). The primers used for amplifying the IL-33 fragment were as follows: sense, 5′ TTAAGGATCCGCCACCATGAGACCTAGAATGAAGTATTCGAAC 3′; and antisense, 5′ TTTTTCTAGATTACATCTTAGAGAGCTTAAACATGATAT 3′. The IL-33 fragments were cloned into the linearized vector (cut by XbaI and BamHI) and ligated with T4 ligase (Takara). The fusion product (pCDH-IL33) was subsequently transformed into competent Stbl3. Clones were selected by ampicillin. The reconstructed plasmids were verified by Sanger sequencing.

For lentiviral production, control vector or pCDH-IL33 with psPAX and pMD2.G were co-transfected into HEK293NT cells. The culture medium was collected at 24 h and 48 h after transfection and then filtered through a 0.45-μm filter before incubation overnight with polyethylene glycol 8000 (PEG 8000) and centrifugation (4000×*g*, 20 min at 4 °C; Thermo). Lentivirus particles were stored at − 80 °C after titration using a titer kit. The ratio of lentivirus-infected MSCs was observed under an inverted fluorescence microscope (Olympus, Tokyo, Japan).

### ELISA

The concentrations of IL-33 and pGE2 in the cell culture supernatant were detected using the corresponding rat enzyme-linked immunosorbent assay (ELISA) kit according to the manufacturer’s instructions (R&D, USA).

### Viability, apoptosis, and proliferation assay

To assess the basic function of MSCs infected with lentivirus, MSC viability, apoptosis, and proliferation were determined using CCK8 (Dojindo Laboratories, Japan), Annexin V-PE/7-AAD (BD, USA), and EdU (5-ethynyl-2′-deoxyuridine, RiboBio, China) respectively, as described previously [4, 10]. Briefly, MSCs in 96-well plates were infected with vector or IL-33 lentivirus for 48 h. Next, the culture medium was changed to fresh medium with 10% CCK8. The absorbance at 450 nm, indicating cell viability, was measured in the Multi-Mode Microplate Reader (BIOTEK, USA). For apoptosis analysis, MSCs were seeded in a 12-well plate and infected with lentivirus for 48 h, and then, the cells were collected before staining with Annexin V-PE for 5 min and 7-AAD for another 5 min. For proliferation assay, MSCs were seeded in a 12-well plate and infected with lentivirus for 48 h, and then, the cells were cultured in EdU medium for 2 h before staining with Alexa Fluor 647 probe. The cells were detected by flow cytometry.

### Co-culture of lymphocytes and MSCs

Mononuclear cells were obtained from spleens using grinding and Ficoll Lymphocyte Separation Medium (Hao Ocean Creatures, China) to isolate lymphocytes according to the manufacturer’s instructions. Then, anti-rat CD4-FITC and separation buffer (Miltenyi Biotec, Germany) were used for the isolation of CD4+ T lymphocytes according to the manufacturer’s instructions. One milliliter of T lymphocytes was seeded in the lower layer of a transwell (concentration 2.5 × 10^4^/ml) with RPMI 1640, 10% FBS, 2.5 μg/ml phytohemagglutinin, and 10 ng/ml IL-2. The differently handled MSCs (MSC, Vector-MSCs, and IL33-MSCs) were seeded in the upper layer of a transwell (3-μm pore size; Costar, USA) and co-cultured for 72 h, and Edu (5-ethynyl-2′-deoxyuridine) cell proliferation kit (RiboBio, China) for proliferation assay was added for another 2 h before T cell proliferation was analyzed using flow cytometry.

### Isolation, differentiation, and co-culture of bone marrow-derived macrophages (BMDMs) with MSCs

Monocytes were isolated from the bone marrow, and erythrocytes were removed with Red Blood Cell Lysis Buffer (Beyotime, China) for 5 min and then washed with RPMI 1640 + 10% FBS three times. The cells were filtered with a 70-μm strainer and cultured for 2 h in RPMI 1640 + 10% FBS before being switched into RPMI 1640 + 10% FBS with 25 ng/ml macrophage colony-stimulating factor (M-CSF, AF-400-28, Peprotech) for 4 days. Then, the differentiated BMDMs were co-cultured with MSCs infected with Vector or IL-33 lentivirus or MSCs only at a 10:1 ratio with a transwell filter (3-μm pore size; Costar, USA). After 48 h of co-culture, the macrophages were collected for flow cytometry analysis of macrophage polarization with macrophage common marker anti-rat CD68 (Bio-Rad, USA), anti-rat iNOS (Abcam, USA) for M1, and anti-rat CD206 (Proteintech, USA) for M2. Flow cytometry assays were conducted using a Millipore Guava® easyCyte 8 (Millipore, USA), and data were analyzed using Guava InCyte™ software.

### Immunofluorescence

Cells were fixed in 4% paraformaldehyde (PFA) for 15 min at room temperature (RT) before permeabilization in 0.1% Triton X-100 for 20 min at RT and blocking with 3% BSA for 1 h. Cells were then incubated with the following primary antibodies: rabbit anti-rat vimentin (SC-5565, Santa Cruz) and goat anti-rat troponin T (SC-8121, Santa Cruz) at 4 °C overnight. Additionally, cells were incubated with the following secondary antibodies: Donkey anti-rabbit-Alexa Fluor 488 (711-545-152, Jackson) and rabbit anti-goat-Alexa Fluor 594 (A-11012, Invitrogen) for 1 h at RT. DAPI was used to counterstain nuclei for 5 min at RT. The immunofluorescence slides were sealed by fluorescence quenching of the tablet and visualized by a Zeiss LSM 880 confocal microscope.

### Surgical model of MI and cell delivery

Acute MI was induced as described previously [10]. In brief, ligation of the left anterior descending (LAD) artery was performed in young male 8-week-old SD rats (~ 250 g) by a single experienced full-time technician. MI was confirmed by myocardial apex blanching. Following MI surgeries, rats were randomized into four groups: received an intramyocardial injection of phosphate-buffered saline (PBS), MSCs infected with empty lentivirus (Vector-MSCs), or MSCs infected with IL-33-overexpressing lentivirus (IL33-MSCs) and the sham group. A total of 1 × 10^6^ passage 3 MSCs were transplanted in the peri-infarct zone at three different sites with a total volume of 20 μl PBS in the free wall of the left ventricle.

To evaluate cell survival of MSCs in vivo, MSCs infected with empty lentivirus (Vector-MSCs) or IL-33-overexpressed lentivirus (IL33-MSCs) were dyed with chloromethylbenzamido (Cell-Tracker TM CM-Dil 11372053; Invitrogen) according to the manufacturer’s instructions, followed by cell transplantation. Rats were anesthetized for MSC survival detection day 3 after MI, and CM-Dil+MSCs were observed via fluorescence microscopy (Olympus, Japan).

### Echocardiographic analysis of the left ventricular function

Echocardiography was performed after 28 days of LAD ligation using the Vevo 2100 system (VisualSonics, Inc., Toronto, ON, Canada) with a 21-MHz probe and MS-250 transducer, which was operated by an investigator blinded to the group designation. Left ventricular end-diastolic diameter (EDD) and end-systolic diameter (ESD) were measured in the short axis and used to calculate LV ejection fraction (LVEF) and LV fraction shortening (LVFS).

### Immune cell assay

To detect the levels of inflammation in rats, spleens and blood were collected at 3 days, 5 days, 7 days, and 14 days after MI surgery. Single-cell suspensions were dissociated by pressing spleen tissue and filtrating cells through a 50-μm nylon mesh and then centrifuging at 1300 rpm for 5 min. Erythrocytes in splenic cells were treated with red blood cell lysis buffer (B541001, Sangon Biotech) for 2 min and washed with PBS three times. Lymphocytes from the blood were collected by lysing erythrocytes with lysis buffer for 3 min × 3 times and washing several times with PBS.

For surface marker anti-rat CD4-FITC (E0074-1633, eBioscience) and anti-rat CD25-APC (380595B, Caltag) staining, cells were incubated with primary antibodies for 30 min at 4 °C. For intracellular protein (anti-rat IFNγ-PE, anti-rat IL-4-PE, anti-rat Foxp3-PE) staining, primary antibodies were washed to remove unbound antibodies and cells were incubated in BD cytofix/cytoperm solution (BD, Bioscience) for 20 min at 4 °C, followed by washing with 1 × BD perm/wash buffer (BD, Bioscience). Subsequently, samples were incubated with secondary antibodies at 4 °C for 30 min in the dark. Flow cytometry assays were conducted using a Millipore Guava® easyCyte 8 (Millipore, USA), and data were analyzed using Guava InCyte™ software.

### Histological examination

Following MI surgery and cell injection, rats were anesthetized with 4% isoflurane (600 ml/min, y0000858, Sigma) and maintained with 2.5% inhalation anesthesia. Hearts from different groups were obtained, fixed in 4% paraformaldehyde (PFA; PH 7.4) and then immersed in optimum cutting temperature compound (OCT; 4583, SAKURA). Serial frozen sections (5 μm thick) were processed via a freezing cryotome (CM1950) for H&E staining and Masson’s trichrome staining (Sigma, USA) to determine the levels of inflammation and collagen deposition in hearts. For each heart, eight to ten sections from apex to base were produced perpendicular to the axis of the left anterior descending coronary artery. The severity of myocardial fibrosis was indicated by the blue staining of collagen deposition. NIH ImageJ software (National Institutes of Health, USA) was used to quantify the cross-section area of fibrosis.

For polarization of macrophage and angiogenic response in heart tissues, immunofluorescence was carried out after unmask antigen with citrate buffer and blocked with 5% BSA for 1 h. Then, the sections were incubated with primary antibodies mouse anti-rat CD68 (Bio-Rad, USA), rabbit anti-rat iNOS (Abcam, USA), rabbit anti-rat CD206 (Abcam, USA), and rabbit anti-CD31 (Abcam, USA) overnight in 4 °C. Subsequently, the sections were incubated with second antibodies Donkey Anti-Mouse-Alexa Fluor 488 (Jackson, USA) and Donkey anti-rabbit-Alex Fluor 594 (Thermo, USA) for 45 min in room temperature. DAPI was used to counterstain nuclei for 5 min at room temperature. The immunofluorescence slides were sealed by fluorescence quenching of the tablet and visualized by a Zeiss LSM 880 confocal microscope.

### Statistical analysis

All experiments were performed at least in triplicate, and statistical analysis was performed using one-way ANOVA for multiple comparisons. Pairwise comparisons were performed using Student’s *t* test. *P* < 0.05 was considered statistically significant. Numeric data (FACS and qPCR) are presented as the mean ± SEM in the figures. GraphPad Prism software (version 5.01; San Diego, CA, USA) was used for statistical analyses.

## Results

### Isolation and identification of MSCs

**Fig. 1:**
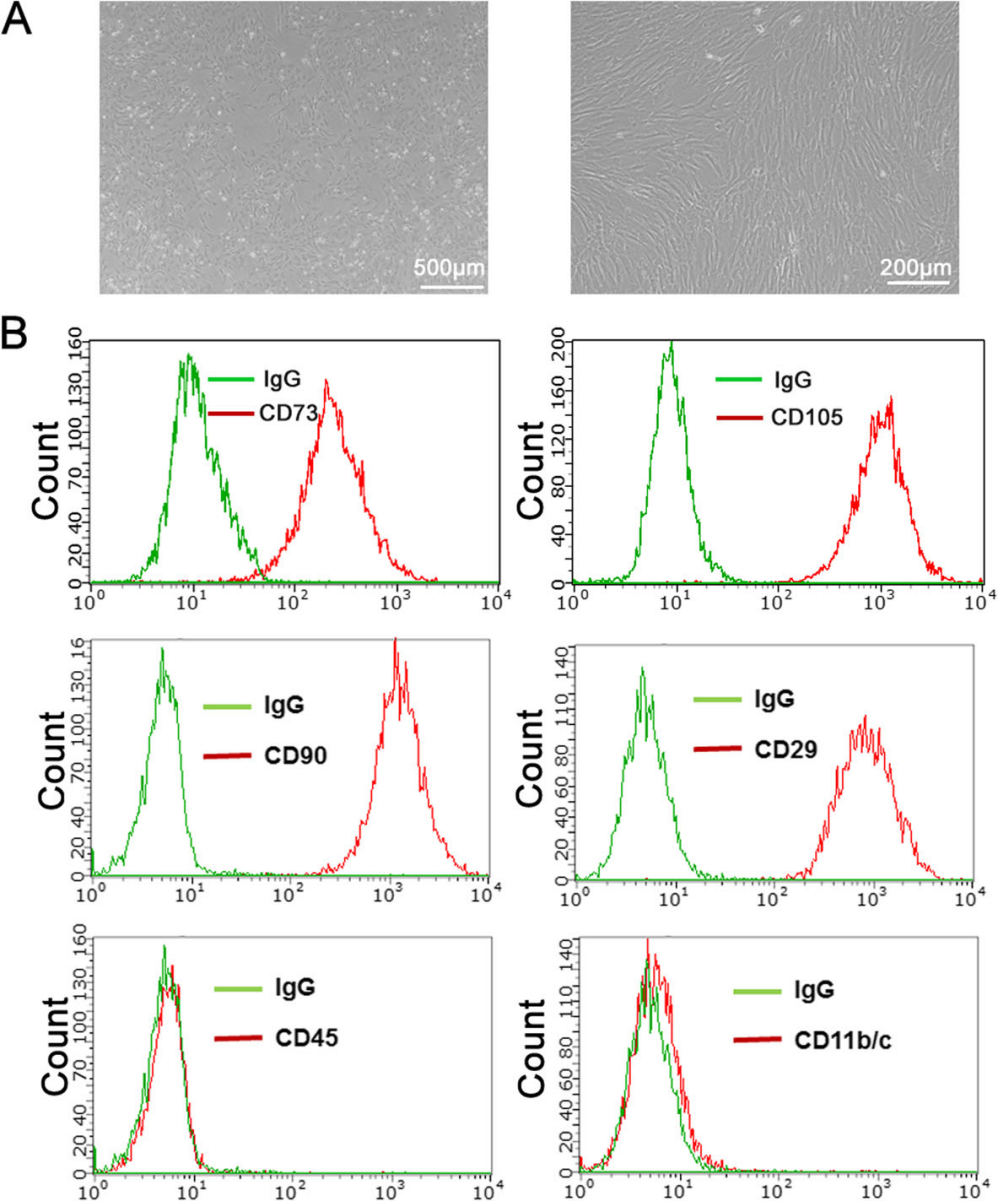
Isolation and identification of MSCs. **a** MSC morphology was observed after 4–6 days of culture. Bar, 500 μm (left), 200 μm (right). **b** Characterization of MSCs by flow cytometry with CD105, CD73, CD90, CD29, CD45, and CD11b/c antibodies (red open histogram). Green open histogram represents the control

To obtain MSCs, we isolated the bone marrow from the femora of 2-week-old SD rats. Typical MSCs were observed after 3 days of culturing with a spindle shape (Fig. 1a). Flow cytometry indicated that MSCs were positive for CD105, CD73, CD90, and CD29 and negative for CD45 and CD11b/c (Fig. 1b), showing typical rat MSC phenotypes.

### Assessment of the IL-33 expression level under hypoxic conditions

**Fig. 2:**
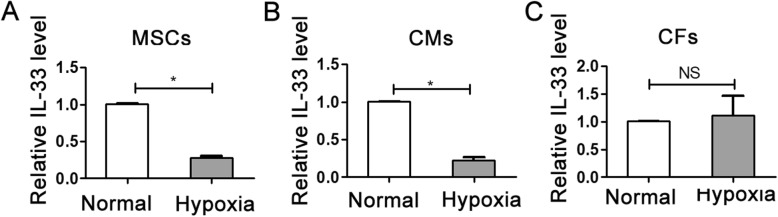
Detection of the IL-33 expression level. Real-time PCR was used to determine the expression of IL-33 in MSCs (**a**), cardiomyocytes (CMs) (**b**), and cardiac fibroblasts (CFs) (**c**). *P < 0.05, NS not significant, as indicated, ANOVA. Each experiment was performed three times

To identify the expression level of IL-33 in CMs, HFs, and MSCs under hypoxic conditions, we first isolated CMs and HFs from neonatal rats. We tested fibroblasts and cardiomyocytes by vimentin for fibroblasts and troponin T for cardiomyocytes (Additional file 1: Figure S1A, B). Then, we tested the IL-33 level using RT-qPCR of MSCs, CMs, and HFs under normal and hypoxic conditions. We found that MSCs and CMs under hypoxic conditions had significantly depressed levels compared with MSCs and CMs under normoxia conditions (Fig. 2a, b) and that HF had no significant difference (Fig. 2c).

### Construction and verification of IL33-MSCs

**Fig. 3:**
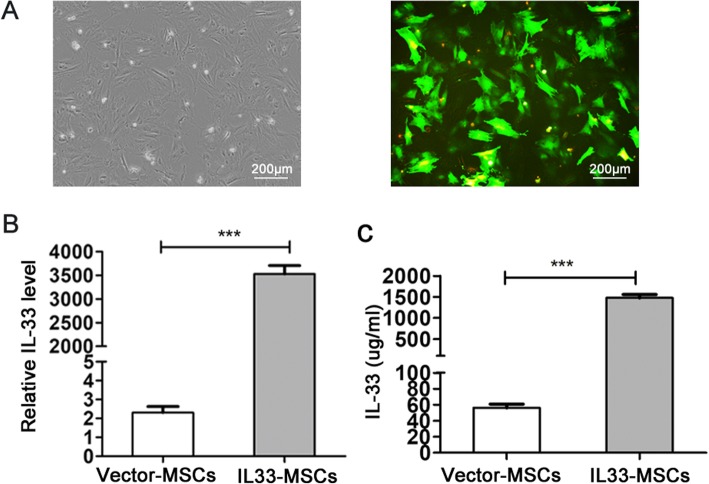
Construction and verification of the plasmid including IL-33. **a** Morphology of MSCs infected with lentivirus observed under an inverted fluorescence microscope (left panel, bright field; right panel, fluorescence field). Bar, 200 μm. Real-time PCR (**b**) and ELISA (**c**) were used to determine the expression of IL-33 in MSCs infected with different lentivirus. ***P < 0.001, as indicated, ANOVA. Each experiment was performed three times

The CDS fragments were cloned into multiple cloning sites (MCS) of pCDH-CMV-MCS-EF1-copGFP using the XbaI and BamHI restriction sites (Additional file 2: Figure S2A). The empty plasmid and the fusion plasmid were co-transfected with a lentivirus package mixed into 293 NT cells. The 293NT cells, which expressed green fluorescent protein in the plasmid, showed successful lentivirus packaging (Additional file 2: Figure S2B). After centrifugation and titering, the MSCs were infected with lentivirus. The MSCs expressing green fluorescent protein (GFP) indicated a successful cell infection (Fig. 3a). The IL-33 levels were determined by quantitative polymerase chain reaction (PCR) and ELISA, and the expression level was elevated significantly, as shown in Fig. 3b, c.

### IL-33 enhanced the survival of MSCs

**Fig. 4:**
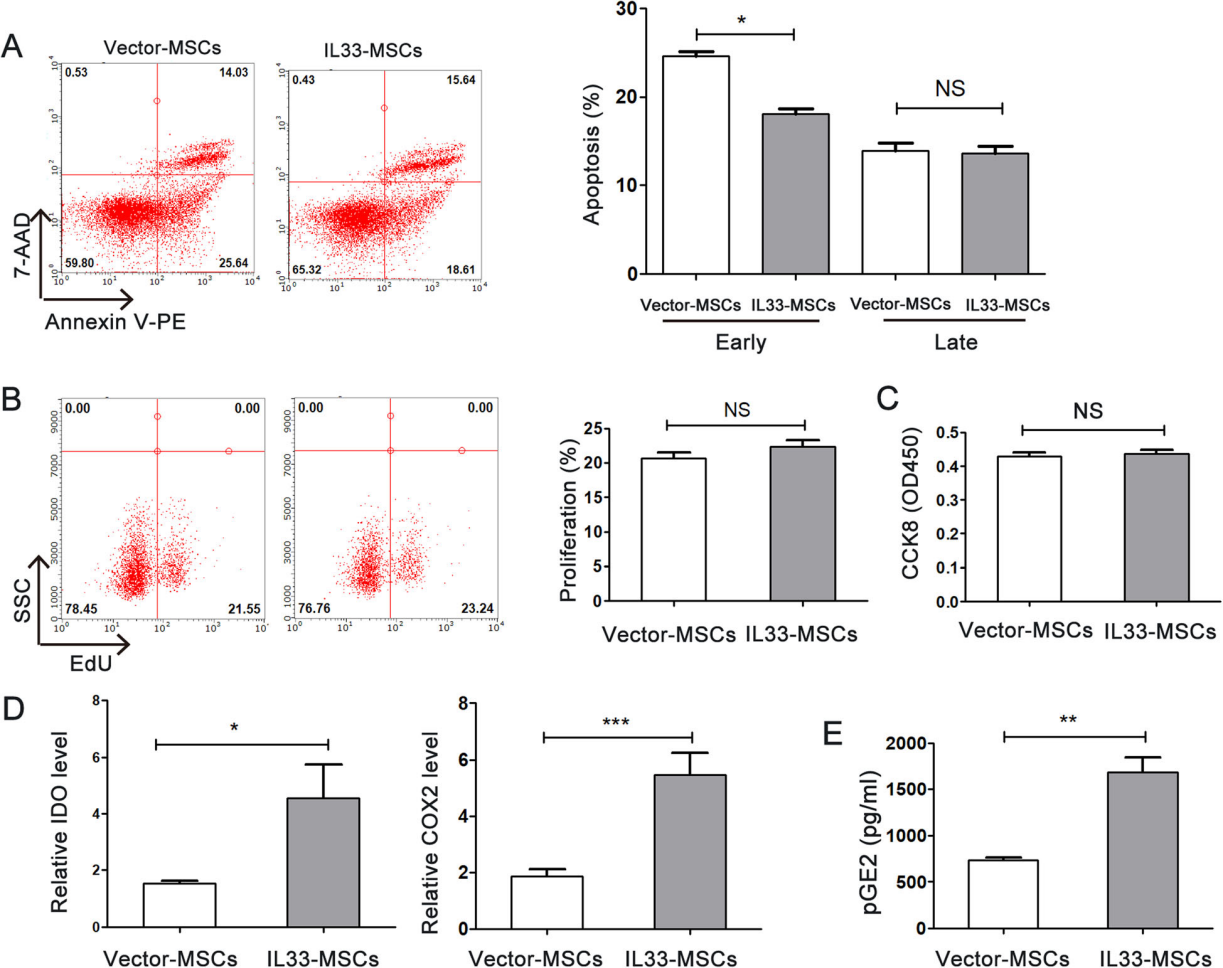
Evaluation of survival, proliferation, and the expression level of immunoregulatory molecules of IL33-MSCs. MSCs were transfected with Vector-MSCs or IL33-MSCs for 48 h. Apoptosis was analyzed by Annexin V-PE/7-AAD staining and flow cytometry. A scatter diagram of apoptosis and histogram of apoptosis are shown in **a**. **b**, **c** IL-33 had no influence on MSC proliferation and viability as determined by EdU and CCK 8 respectively (*n* = 6). **d** Real-time PCR was used to determine the expression of IDO and COX2 in MSCs transfected with different plasmids (*n* = 4). **e** ELISA was used to determine the protein level of pGE2 in MSCs transfected with different plasmids (*n* = 4). *P < 0.05, ***P < 0.001, as indicated, ANOVA. Each experiment was performed three times

Viability and apoptosis were used as criteria to evaluate the capacity of IL-33 to improve cell survival. To identify the potential pro-survival function of IL-33 on MSCs, we tested the cell viability, apoptosis, and proliferation of MSCs after lentivirus infection. After infection with lentivirus for 24 h, MSCs were switched to serum-free and hypoxic conditions for another 24 h and then stained with Annexin V-PE (for early apoptosis) and 7-AAD (for late apoptosis). Cell viability and proliferation was tested by CCK8 and EdU. We found that IL-33 reduced early MSC apoptosis (Fig. 4a) and had no significant influence on MSC viability and proliferation (Fig. 4b, c).

### IL-33 elevated the immunoregulatory capacity of MSCs

We first evaluated the expression level of indoleamine-2, 3 dioxygenase (IDO) and cyclooxygenase (COX)-2, two important genes for the immunoregulatory capacity of MSCs. Our data showed a significant elevation of the expression levels of both genes (Fig. 4d). Prostaglandin (PG) E2 protein level is influenced by COX2 and influences the immunoregulatory of MSCs which was elevated significantly in IL33-MSCs group compared with Vector-MSCs group (Fig. 4e).

**Fig. 5:**
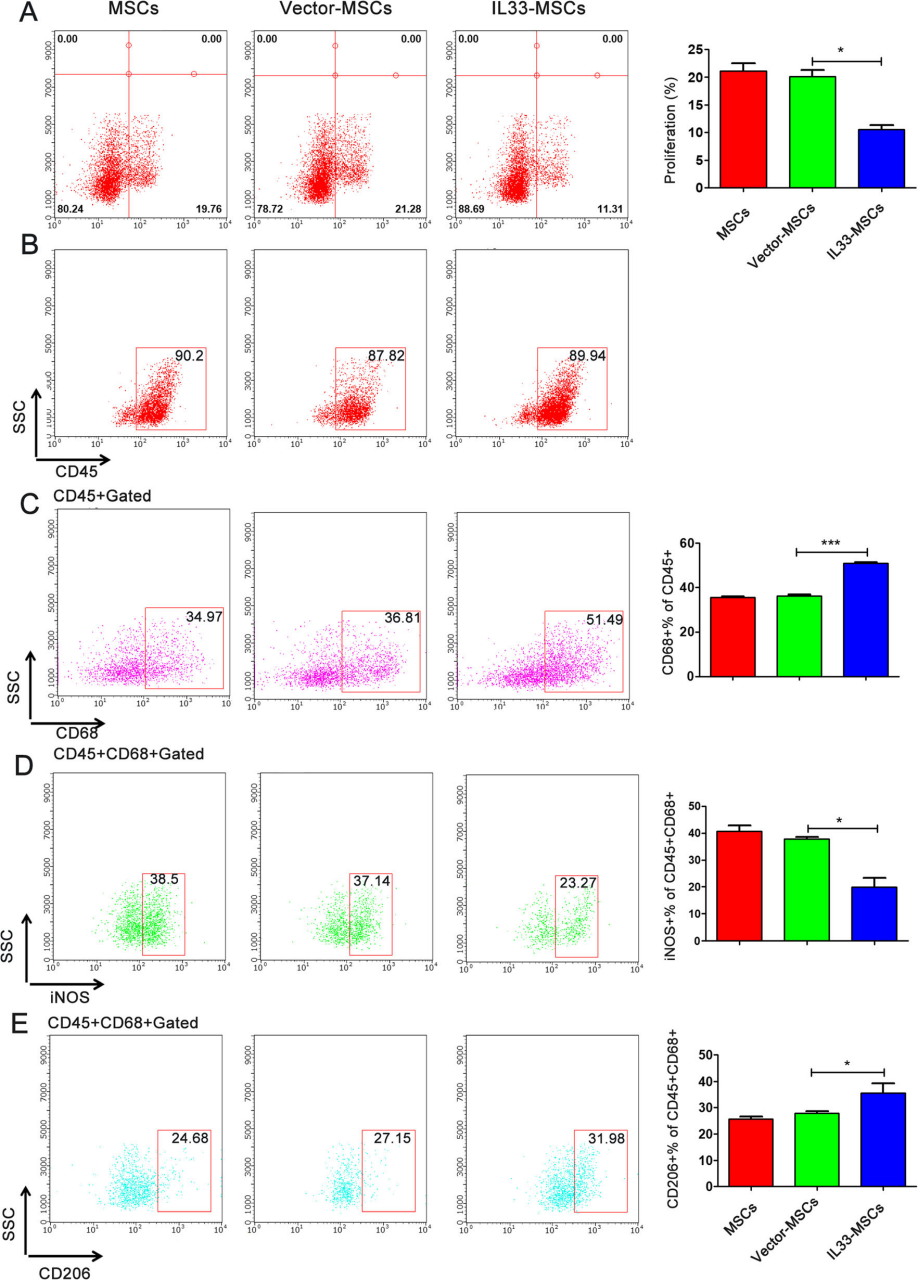
IL33-MSCs alter T cell proliferation and macrophage M1/M2 polarization in vitro. **a** Proliferation of T cells was measured by EdU+ using flow cytometry. **b** The percent of CD45+ immune cells used for macrophage polarization analysis. **c** The flow cytometry plots and analyses of the percentages of CD68+ macrophages and the M1 marker iNOS (**d**) and M2 marker CD206 (**e**) in cells co-cultured with different MSCs. *P < 0.05, ***P < 0.001, as indicated, ANOVA. Each experiment was performed three times

To study T cell proliferation, CD4^+^ T cells were co-cultured with MSCs infected with different viruses or no virus. After 72 h of culture, T cell proliferation analysis as assessed by EdU showed a low proliferation rate in the IL33-MSCs group (Fig. 5a).

Macrophages are important immune cells that have two subtypes. MSCs treated with IL-1β or exosome influenced macrophage polarization [8]. We investigated whether IL33-MSCs influenced macrophage polarization, which has an important effect on the immunoregulation of the early stage of ischemia-reperfusion injury. After co-culturing of macrophages with IL33-MSCs, Vector-MSCs, or MSCs for 48 h, flow cytometry analysis was performed with CD68 and CD206 for type II macrophages and CD68 and iNOS for type I macrophages. The results showed that macrophages induced from bone marrow monocytes co-cultured with IL33-MSCs expressed higher percent of CD68 and CD206 and lower percent of CD68 and iNOS versus the MSCs or Vector-MSCs groups (Fig. 5b–e).

### Assessment of the inflammation level at different times following myocardial infarction

**Fig. 6:**
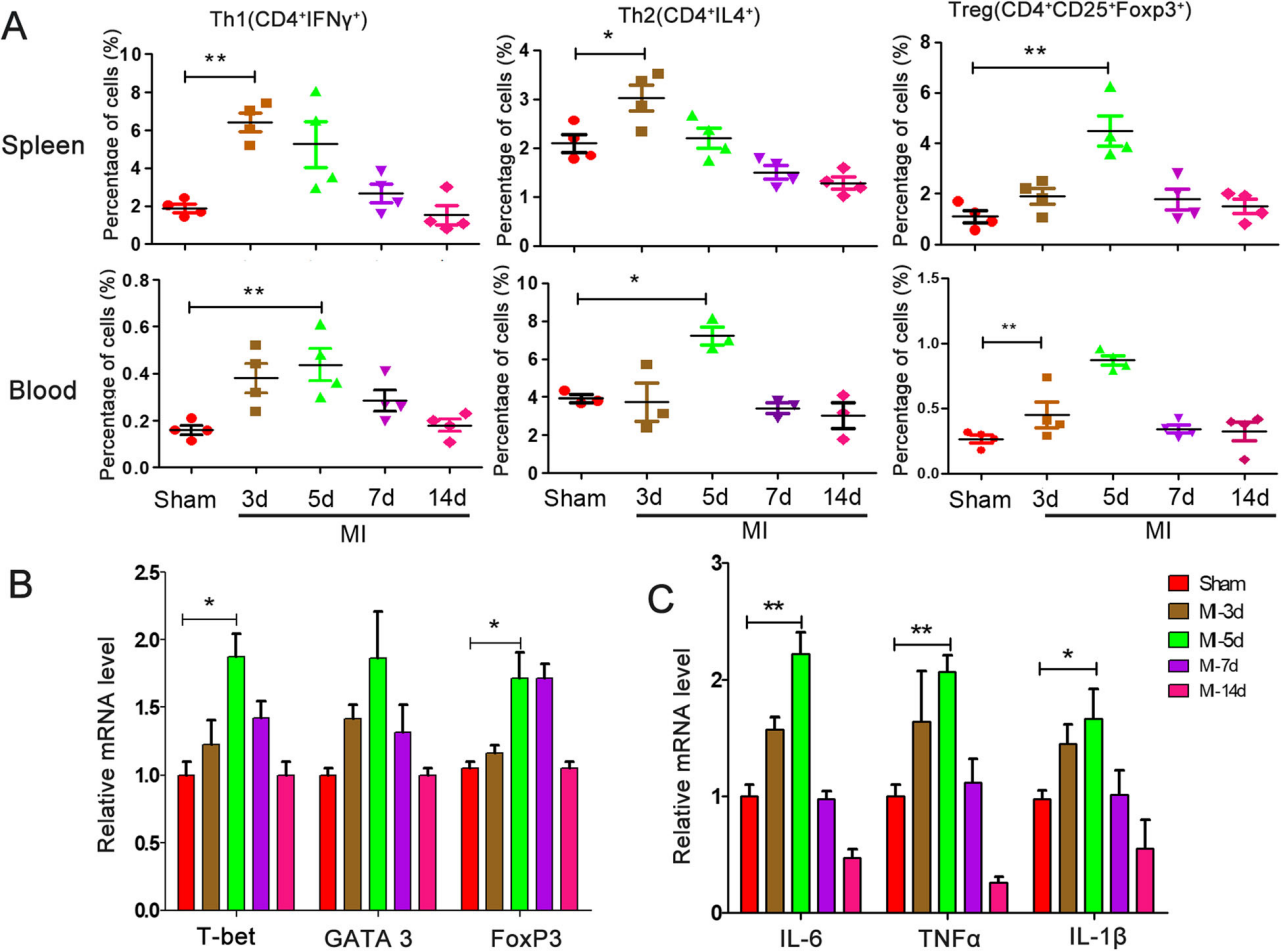
Inflammatory level at different time points after MI. Rats were sacrificed at different times following MI surgery. **a** Flow cytometry analysis of T cell populations and subtype polarization from the spleen and blood 3, 5, 7, and 14 days after MI. Real-time PCR showed transcription factors of T cell subtype (**b**) and inflammatory factors (**c**) from the heart border and infarct zone, *n* = 4. *P < 0.05, **P < 0.01, ***P < 0.001, as indicated, ANOVA. Each experiment was performed three times

To examine the time point at which the most serious inflammation occurred, rats underwent LAD ligation and were sacrificed at day 3, day 5, day 7, and day 14 after MI (Additional file 3: Figure S3). T cells with different subtypes have significant roles in immunoregulation in systemic and local inflammation. Therefore, we tested the subtypes of Th1 (CD4^+^IFNγ^+^), Th2 (CD4^+^IL4^+^), and Treg (CD4^+^CD25^+^Foxp3^+^) cells, which influence inflammation associated with heart disease (Fig. 6a). The results showed that inflammation was robust at days 3 and 5 after MI both in the spleen and blood. Th1 cells were significantly higher than Sham; Th2 and Treg cells were the highest at day 5. The expression levels of the transcription factors T-bet, GATA3, and Foxp3 relative to the differentiation of CD4^+^ T cells from the infarct and border zones were consistent with the corresponding cell numbers (Fig. 6b). Cytokines are also markers of inflammation levels, and expression of IL-6, TNFα, and IL-1β from the infarct and border zones was the highest at day 5, which confirmed the time point of robust inflammation (Fig. 6c).

### Cardiac injection of IL33-MSCs was used to evaluate heart functional recovery following myocardial infarction

**Fig. 7:**
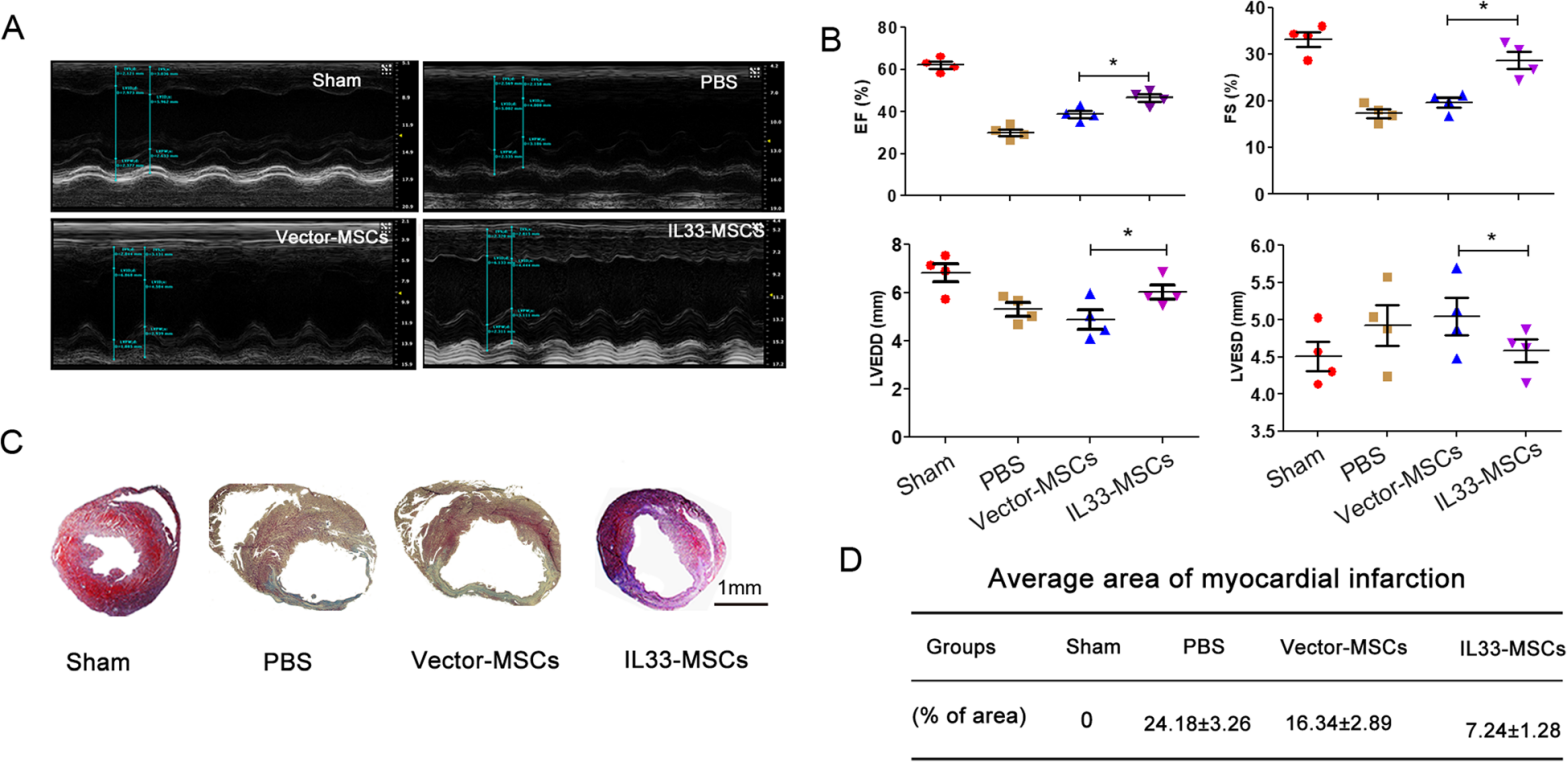
Echocardiography and histological analysis of heart function. **a** Representative echocardiography of rats following the PBS, Vector-MSCs and IL33-MSC injection for 28 days. **b** Quantitative analysis of the left ventricular ejection fraction (LVEF), left ventricular fractional shortening (LVFS), left ventricular end-diastolic diameter (LVEDD), and left ventricular end-systolic diameter (LVESD) in different groups. **c** Representative Masson trichrome staining of the explanted heart showed a reduced fibrosis level in the IL33-MSC injection group (*n* = 4). Bar, 1 mm. **d** Percentage of the fibrotic area calculated and averaged by ImageJ software (*n* = 4)

To assess the efficacy of IL33-MSCs in the rat MI model, rats underwent LAD ligation and an intramyocardial injection near the ligation site in the free wall of the heart with PBS, Vector-MSCs, IL33-MSCs, and Sham, which was used as a control group. Echocardiography was performed 28 days after MI, and the representative images of M-Mode are shown in Fig. 7a. The left ventricular ejection fraction and fractional shortening were used as major in vivo indexes of heart function, and the results showed that IL33-MSCs significantly elevated the EF and FS versus in the Vector-MSCs group (*P* < 0.05). Left ventricular end-diastolic diameter and left ventricular end-systolic diameter were improved in the IL33-MSCs group compared with the Vector-MSCs group (Fig. 7b).

Fibrosis and remolding are the secondary stage after MI and can lead to a further reduction of cardiac function. Therefore, we evaluated the extent of fibrosis with Masson trichrome staining 28 days after MI and with different MSC injections. As illustrated in Fig. 7C, the normal myocardium was stained red, whereas the blue color indicated fibrosis tissue. The fibrosis area was analyzed by ImageJ software and showed that IL33-MSCs significantly reduced the fibrosis area compared with both the PBS and Vector-MSCs groups (Fig. 7d).

### Ex vivo histological examination of the inflammation level and macrophage polarization

**Fig. 8:**
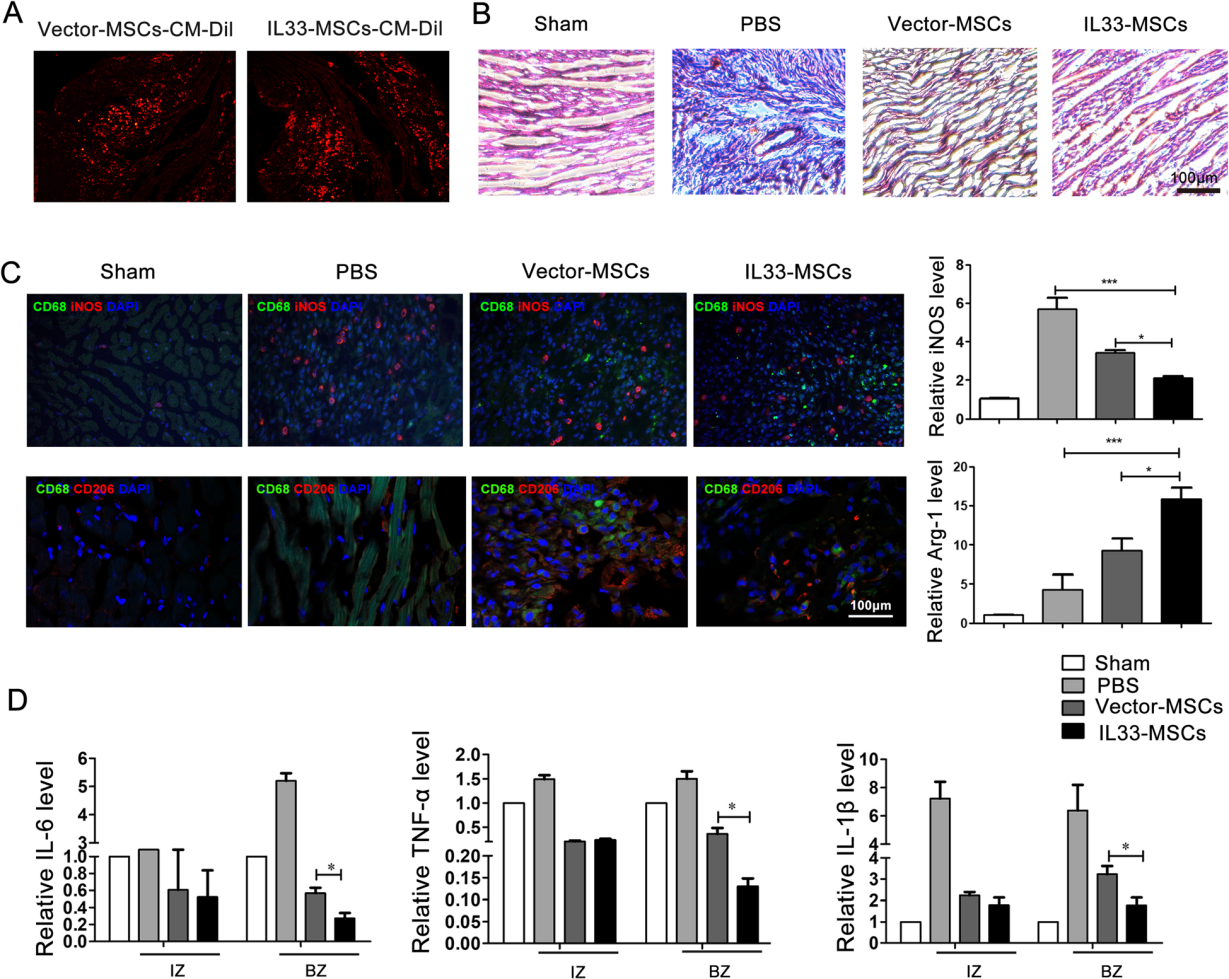
Histological examination of the inflammation level. **a** Fluorescent photographs of surviving MSCs with red fluorescence in vivo. **b** Representative H&E staining of explanted hearts at 5 days after MI showed a reduced inflammatory level in the IL33-MSCs group (*n* = 4). Bar, 100 μm. **c** Representative immunofluorescence staining of macrophages, the M2 marker CD68 (green) and CD206 (red), and the M1 marker CD68 (green) and iNOS (red). Bar, 100 μm. **d** Real-time PCR analysis of inflammatory factor in infarct zone and bore zone of hearts with different MSC injection. *n* = 4, *P < 0.05, as indicated, ANOVA

Cell survival and migration are important in the therapeutic effect of MSCs in vivo. We therefore assessed the survival and retention of MSCs in the heart tissue by counting CM-Dil+ cells, which indicate the viable exogenous MSCs. The results showed more survival and retentional MSCs in the heart tissue in IL33-MSCs group (Fig. 8a). For histological analysis, hearts from different groups were collected at day 5 after MI, which was the time point of robust inflammation. H&E staining indicated a reduced infiltration of inflammatory cells in the border zone of MI for the IL33-MSCs group compared with PBS and Vector-MSCs (Fig. 8b). A growing body of evidence has demonstrated that macrophages have a strong influence on the level of inflammation in the heart at day 5. To detect the effects of IL33-MSCs on macrophage polarization and inflammation, we measured the expression of known macrophage markers in the peri-infarct tissue of the PBS-, Vector-MSC-, and IL33-MSC-treated hearts. The protein markers CD68 and CD206 are expressed by M2 macrophages and can be induced to repress inflammation; however, CD68 and iNOS are expressed by M1 macrophages and increase inflammation. Immunostaining of heart slides in the border zone of the MI confirmed that IL33-MSCs reduced inflammation in the heart, increasing the expression of CD206 and decreasing the expression of iNOS (Fig. 8c). Furthermore, we found that inflammation-related genes were reduced in the border zone of rat hearts from the IL33-MSCs group, including IL-6, TNFα, and IL-1β, but no differences were observed in the infarct zone (Fig. 8d), which used the whole heart of the sham as control. Excepting the influence on inflammatory regulation, we found the expression of CD31 was also elevated in the IL33-MSCs group compared with the PBS group (Additional file 4: Figure S4). Therefore, we concluded that the IL33-MSC injection reduced the inflammatory level in hearts with myocardial infarction.

## Discussion

In this study, we found that the expression level of IL-33 was reduced in MSCs and cardiomyocytes under hypoxic conditions, which indicated that IL-33 had an effect on MI within an ischemic anoxia environment. The expression level of IL-33 under hypoxic condition seems inconformity in different cells and tissues, which may be regulated by a different manner [18, 19]. Therefore, we constructed a plasmid overexpressing IL-33 to test the therapeutic effect of IL-33.

Mesenchymal stem cells (MSCs) are a class of stem cells that have important effects on the regulation of inflammation and have been used in many disease treatments and clinical trials. A growing number of studies have shown that many factors can enhance the regulatory and therapeutic functions of MSCs derived from different tissues [7, 20]. To combine the application of IL-33 and MSCs, we used packaged lentivirus with plasmids overexpressing IL-33 to infect MSCs. We found that overexpression of IL-33 in MSCs had no side effects of the basic functions of MSCs, such as apoptosis and cell viability. Interestingly, MSCs co-cultured with spleen monocytes and bone marrow-derived monocytes demonstrated that MSCs transduced with IL-33 (IL33-MSCs) had a significantly better immunosuppression ability compared with Vector-MSCs. In vitro co-culture experiment demonstrated that IL33-MSCs induced more percent of CD68+ cells and enhanced the percent of CD68+CD206+M2 macrophage (Fig. 5c–e). Moreover, we found more co-stain of CD68 and CD206 in the tissue of IL33-MSCs group compared with Vector-MSCs group in vivo (Fig. 8c). In accordance with the previous result, it was concluded that the cytokine IL-33 enhanced MSC regulation of inflammation.

In an in vivo study, we constructed an acute MI model, and under treatment with differently handled MSCs (Vector-MSCs or IL33-MSCs), echocardiography and Masson trichrome staining experiments indicated that overexpression of IL-33 in MSCs could enhance the left ventricular ejection fraction and fractional shortening and reduce heart tissue fibrosis (Fig. 7a–d). The result is in accordance with a previous study [21] in IL-33^−/−^ mice with transverse aortic constriction (TAC) surgery. Both of the two studies highlighted the cardioprotective role of IL-33 in heart disease.

Due to the close links between the inflammation stage and repair stage, non-selective inhibition of inflammation after MI may have detrimental effects on scar formation and accentuation of adverse remolding [22]. Therapeutic modulation of the inflammatory response may have promise for the prevention of post-infarction heart failure. Cardiomyocytes undergo massive sudden necrosis following MI as well as secrete cytokines and drive the infiltration of inflammatory cells, including neutrophils, monocytes (Ly6C^hi^), lymphocytes (T cells and B cells) [23–26], mast cells, and macrophages. At the early stage of myocardial infarction, except neutrophil infiltration of the infarct area, which is part of the early inflammatory response after MI [27], macrophages have important effects on inflammation regulation in MI. Macrophages are heterogeneous and are from two different pools: the CCR2 subset, which is an embryonically established lineage that originates from the yolk sac and fetal monocytes, and circulating CCR2^+^ monocytes [28, 29]. In the MI model, the resident cardiac macrophages died and are replaced by monocyte-derived CCR2^−^ cells. In the first stage, Ly6C^hi^ monocytes and M1 macrophages with high expression of proteinase and TNF have a pro-inflammatory function; in the second stage, Ly6C^low^ monocytes and M2-like macrophages with high expression of IL-10, TGF-β, and VEGF have an anti-inflammatory function [30]. In this study, we found that IL33-MSCs could enhance the polarization of M2 macrophages (Figs. 5 and 8b) in vitro and in vivo, in accordance with previous reports that indicated that macrophages have therapeutic effects on myocardial healing [31]. CD4^+^Foxp3^+^Treg modulates macrophage differentiation toward the M2 phenotype [32]. We also found that CD4+Foxp3+Tregs increased following induction of the MI model (Fig. 6a).

One troublesome problem in the field of clinical MSCs is that complement inhibition does not reduce mortality and major adverse events in patients with MI and acute coronary syndromes [33, 34]. There are many reasons to contribute to adverse clinical outcomes: (1) inflammatory mediators are pleiotropic, showing a wide range of actions on different cells; (2) temporal and spatial aspects are critical factors for therapy; (3) the spatial heterogeneity of the cellular environment in vivo is complex; and (4) genetic background, concomitant condition, age, and gender all influence the outcome of therapy.

## Conclusion

In summary, we successfully modified MSCs with IL-33-overexpressing plasmids, and the modified IL33-MSCs enhanced the function of the heart under LAD surgery. We also found that macrophages had important effects in the recovery of heart function (Graphical Abstract).

## Additional files

Additional file 1: **Figure S1.** Identification of fibroblasts and cardiomyocytes isolated from neonatal rats. (A) Characterization and identification of fibroblasts immunostained with vimentin. (B) Characterization and identification of cardiomyocytes immunostained with Troponin T. Bar, 200 μm. DAPI is used for stain of nucleus (Blue). (TIF 3323 kb)

Additional file 2: **Figure S2.** Construction and package of plasmid overexpressing IL-33. (A) Construction and restriction enzyme cutting sites of the plasmid pCDH-IL33. (B) Fluorogram of the packaged lentivirus including IL-33 in HEK293NT cells. Bar, 500 μm. (TIF 1857 kb)

Additional file 3: **Figure S3.** Schematic diagram for time point study. Rats that underwent MIsurgery were sacrificed at different time points. (TIF 2232 kb)

Additional file 4: **Figure S4.** Representative immunofluorescence staining of endothelial cells, the marker CD31 (Green). Bar, 100 μm. (TIF 16279 kb)

## Data Availability

The datasets used and/or analyzed during the current study are available from the corresponding author on reasonable request.

## Abbreviations

BMDM: Bone marrow-derived monocyte-macrophages
IL-33: Interleukin 33
LVEF: Left ventricular ejection fraction
LVFS: Left ventricular fractional shortening
MSCs: Mesenchymal stem cells
