# Supplementary figures and images for: The enhanced effect and underlying mechanisms of mesenchymal stem cells with IL-33 overexpression on myocardial infarction
Source: Stem Cell Res Ther. 2019 Sep 23;10:295. doi: 10.1186/s13287-019-1392-9 (PMC6757387; doi:10.1186/s13287-019-1392-9)

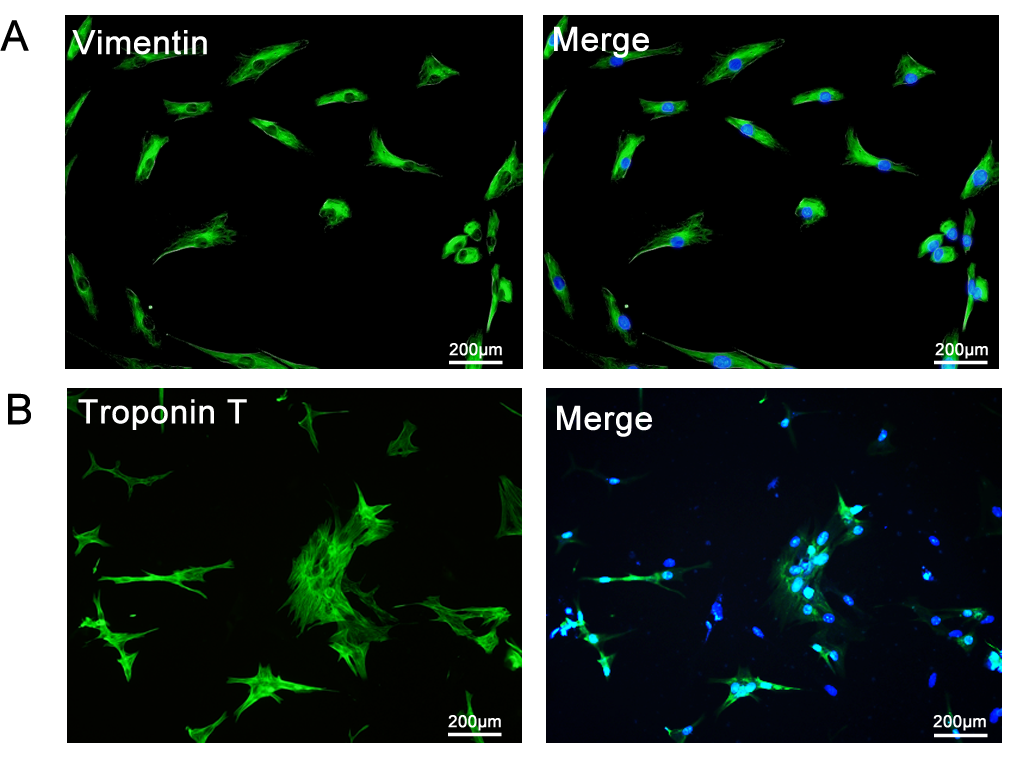

Supplement: Supplementary file 1 — Figure S1. Identification of fibroblasts and cardiomyocytes isolated from neonatal rats. (A) Characterization and identification of fibroblasts immunostained with vimentin. (B) Characterization and identification of cardiomyocytes immunostained with Troponin T. Bar, 200 μm. DAPI is used for stain of nucleus (Blue). (TIF 3323 kb) [file 13287_2019_1392_MOESM1_ESM.tif]

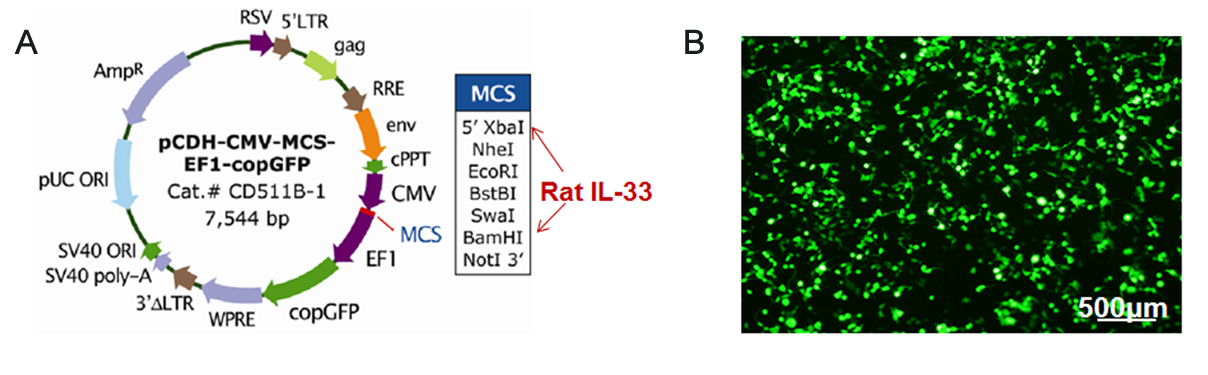

Supplement: Supplementary file 2 — Figure S2. Construction and package of plasmid overexpressing IL-33. (A) Construction and restriction enzyme cutting sites of the plasmid pCDH-IL33. (B) Fluorogram of the packaged lentivirus including IL-33 in HEK293NT cells. Bar, 500 μm. (TIF 1857 kb) [file 13287_2019_1392_MOESM2_ESM.tif]

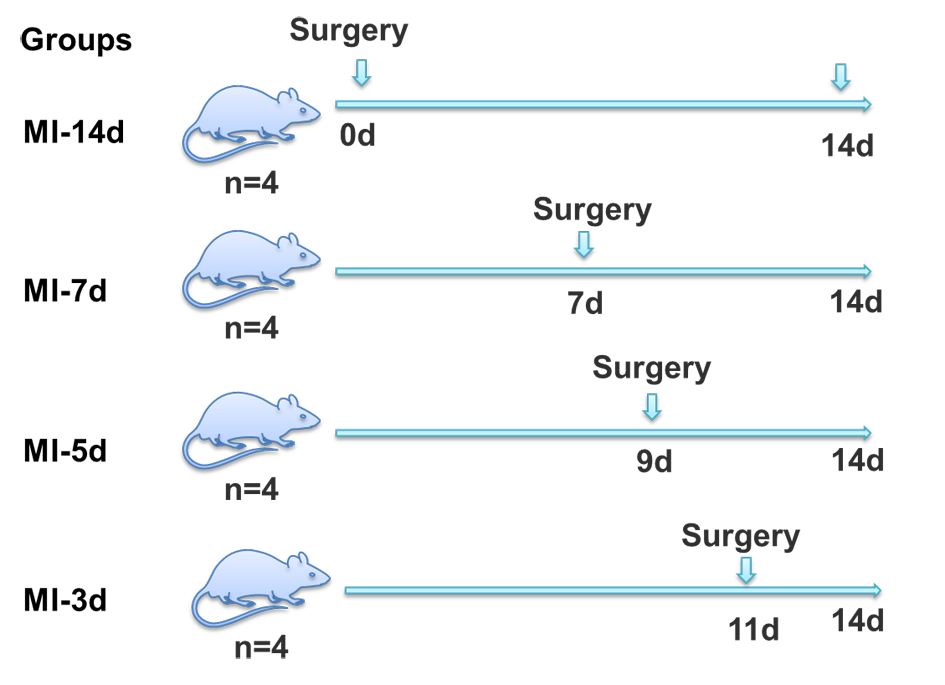

Supplement: Supplementary file 3 — Figure S3. Schematic diagram for time point study. Rats that underwent MIsurgery were sacrificed at different time points. (TIF 2232 kb) [file 13287_2019_1392_MOESM3_ESM.tif]

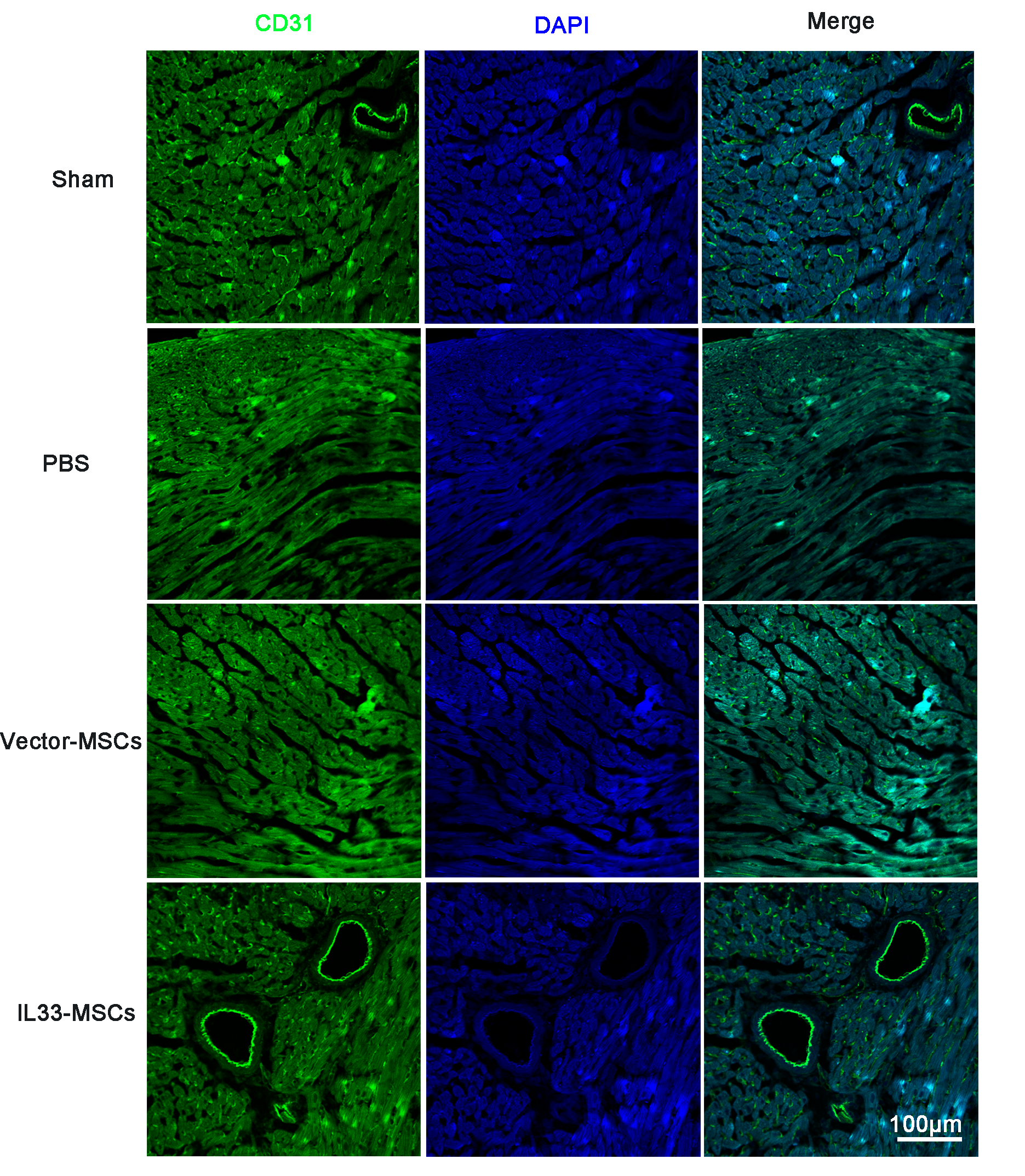

Supplement: Supplementary file 4 — Figure S4. Representative immunofluorescence staining of endothelial cells, the marker CD31 (Green). Bar, 100 μm. (TIF 16279 kb) [file 13287_2019_1392_MOESM4_ESM.tif]
